# Analysis of anterior trunk symmetry index (ATSI). Preliminary report

**DOI:** 10.1186/1748-7161-8-S1-O25

**Published:** 2013-06-03

**Authors:** L Stolinski, T Kotwicki, D Czaprowski, J Chowanska

**Affiliations:** 1 Rehasport Clinic, Poznan, Poland; 2 Spine Disorders Unit, Department of Pediatric Orthopedics and Traumatology, University of Medical Sciences, Poznan, Poland; 3 The Faculty of Physiotherapy, Jozef Rusiecki University, Olsztyn, Poland

## Background

Spinal deformities and postural disorders can be assessed by evaluation of trunk surface deformity. Usually, the back shape is assessed. However, the anterior trunk can also develop deformity, which is observed by the patient more easily than the back surface.

## Aim of the study

To introduce a new parameter, Anterior Trunk Symmetry Index (ATSI), for anterior trunk deformity assessment.

## Methods

Seventy primary school children, free of idiopathic scoliosis, both sexes, aged 6-7 years, mean 6.9 ± 0.3 years were examined with digital photography of their trunk, taken from the front in standardized conditions. The anatomical landmarks were: sternal notch, acromions, axilla folds, waist lines, and umbilicus. ATSI was defined as the sum of six indices: three frontal plane asymmetry indices (one for sternal notch, axilla folds and waist lines, respectively) and three height difference indices, (one for acromions, axilla folds, and waist lines, respectively). The software was developed for semi-automatic calculation of ATSI, after the anatomical points are indicated on a digital photo by the observer. The intra-observer error was calculated by the first author, by measuring four times the pictures of 20 children, in the interval of at least one day. The inter-observer error was calculated by one surgeon, and three experienced physiotherapists, by measuring the pictures of 20 children. The normal value limit was calculated as mean ± 3SD.

## Results

The assessment of the ATSI on digital photography took around 1 minute. The mean ATSI value for 70 children was 22.6 ± 10.8. The intra-observer error was 1.23. The inter-observer error for the four observers was 3.08. The normal value limit was 32.3.

## Conclusion

This new surface parameter can be easily calculated on regular digital photographs of the anterior trunk. Both intra- and inter-observer errors are small, indicating possible reliability of ATSI for the assessment of anterior trunk asymmetry in children. Further studies are needed to assess the clinical usefulness of the ATSI parameter.
